# Role and potential of ^18^F-fluorodeoxyglucose-positron emission tomography-computed tomography in large-vessel vasculitis: a comprehensive review

**DOI:** 10.3389/fmed.2024.1432865

**Published:** 2024-08-07

**Authors:** Javier Collada-Carrasco, Nieves Gómez-León, Valentina Castillo-Morales, Blanca Lumbreras-Fernández, Santos Castañeda, Víctor Rodríguez-Laval

**Affiliations:** ^1^Department of Radiology, Hospital Universitario de La Princesa, Autonomous University of Madrid, IIS-Princesa, Madrid, Spain; ^2^Department of Nuclear Medicine, Hospital General Universitario Gregorio Marañón, Madrid, Spain; ^3^Department of Rheumatology, Hospital Universitario de La Princesa, Autonomous University of Madrid, IIS-Princesa, Madrid, Spain

**Keywords:** PET-CT, large-vessel vasculitis, giant cell arteritis, polymyalgia rheumatica, Takayasu’s arteritis

## Abstract

Large-vessel vasculitis (LVV) is a group of diseases characterized by inflammation of the aorta and its main branches, which includes giant cell arteritis (GCA), polymyalgia rheumatica (PMR), and Takayasu’s arteritis (TAK). These conditions pose significant diagnostic and management challenges due to their diverse clinical presentations and potential for serious complications. ^18^F-fluorodeoxyglucose positron emission tomography-computed tomography (^18^F-FDG-PET-CT) has emerged as a valuable imaging modality for the diagnosis and monitoring of LVV, offering insights into disease activity, extent, and response to treatment. ^18^F-FDG-PET-CT plays a crucial role in the diagnosis and management of LVV by allowing to visualize vessel involvement, assess disease activity, and guide treatment decisions. Studies have demonstrated the utility of ^18^F-FDG-PET-CT in distinguishing between LVV subtypes, evaluating disease distribution, and detecting extracranial involvement in patients with cranial GCA or PMR phenotypes. Additionally, ^18^F-FDG-PET-CT has shown promising utility in predicting clinical outcomes and assessing treatment response, based on the correlation between reductions in FDG uptake and improved disease control. Future research should focus on further refining PET-CT techniques, exploring their utility in monitoring treatment response, and investigating novel imaging modalities such as PET-MRI for enhanced diagnostic accuracy in LVV. Overall, ^18^F-FDG-PET-CT represents a valuable tool in the multidisciplinary management of LVV, facilitating timely diagnosis and personalized treatment strategies to improve patient outcomes.

## 1 Introduction

Large-vessel vasculitis (LVV) encompasses a group of diseases characterized by inflammation of the vessel wall of the median and great arteries (aorta and main branches), giving rise to systemic inflammation and territorial ischemia. The most characteristic entities are giant cell arteritis (GCA), polymyalgia rheumatica (PMR) and Takayasu’s arteritis (TAK). Nevertheless, inflammation of the wall of median and large blood vessels can be detected in other systemic inflammatory and autoimmune diseases, such as spondyloarthritis, relapsing polychondritis, Behçet’s disease or IgG_4_-related disease.

In this review, we will focus on the role of ^18^F-fluorodeoxyglucose-positron emission tomography-computed tomography (^18^F-FDG-PET-CT) in the diagnosis and follow-up of GCA, PMR and TAK.

## 2 Discussion

### 2.1 Large-vessel vasculitis

Giant cell arteritis (GCA) is the most common vasculitis in individuals over the age of 50 years in Northern Europe (1); the number of GCA patients in Europe, North America and Oceania is expected to be greater than three million by 2050 (2). A recent study carried out in Spain estimated an annual incidence of 7.42 cases per 100,000 people with age ≥ 50 years, with a peak for patients aged 80–84 years. Furthermore, the incidence was greater in women (10.06) than in men (4.83) (3).

GCA includes two main and opposed phenotypes: cranial GCA (C-GCA) and large-vessel GCA (LV-GCA). PMR is considered a part of the GCA disease spectrum by some authors (4, 5). C-GCA patients exhibit headaches, changes in vision or jaw claudication; whereas, at the other end of the spectrum, PMR causes inflammatory musculoskeletal manifestations such as arthritis, bursitis, and tenosynovitis (6). Both typically affect people over 70 years, while LV-GCA tends to appear earlier (4).

These phenotypes frequently overlap, as a third of C-GCA patients show extra-cranial involvement and 10–40% of PMR patients also experience C-GCA or LV-GCA (7). In addition, more than a quarter of PMR patients may have subclinical GCA (8), although meta-analyses have failed to identify a specific marker for their early identification. Therefore, we consider necessary a paradigm shift in the assessment of PMR patients that favors the early implementation of imaging studies.

Takayasu’s arteritis (TAK) is a rare autoimmune granulomatous condition of the aorta and primary branches, including the carotid, subclavian, renal, ilio-femoral and splanchnic arteries. Coronary involvement occurs in 15 to 25% of cases. Its incidence is approximately 1 case/million people/year, with a higher prevalence among Asian population and younger women. TAK presents two clinical phases that may overlap: an acute/systemic phase with constitutional symptoms caused by active inflammation, which can last for years before the definitive diagnosis (9); and a chronic/occlusive phase characterized by structural vascular abnormalities. Intimal hyperplasia, observed in over 90% of the cases, leads to stenosis or occlusion, while aneurysms occur in approximately 25% of the cases. Symptoms of the occlusive phase include weakened peripheral pulses, claudication, and differences in blood pressure between arms. As previously stated, diagnosis often occurs late in the disease course.

^18^F-FDG-PET-CT plays a vital role in visualizing blood vessel involvement, disease extension and activity in patients with established or suspected LVV (10, 11). It is useful to confirm the diagnosis when LV-GCA or TAK is suspected based on clinical and paraclinical findings. In cases of suspected C-GCA, ^18^F-FDG-PET-CT allows assessing large artery involvement, particularly with digital PET scanners (12). The addition of ^18^F-FDG-PET-CT to diagnosis assessment in suspected GCA cases improves diagnostic accuracy and prompts therapy changes in approximately a quarter of patients (11). This technique is also useful to confirm or rule out large-vessel inflammation and assess musculoskeletal involvement in established or suspected PMR. Additionally, ^18^F-FDG-PET-CT aids in differentiating PMR from other musculoskeletal diseases in the elderly, such as rheumatoid arthritis or late-onset spondyloarthritis (6, 13). Furthermore, LVV or PMR may be identified in ^18^F-FDG-PET-CT studies conducted for fever or inflammation of unknown origin.

### 2.2 Scanning protocol

For optimal performance standardizing ^18^F-FDG-PET-CT scans is imperative, including the entire procedure, patient preparation, acquisition, reconstruction, and analysis.

Patients are advised to fast for a minimum of 6 h and abstain from strenuous activity for 24 h before ^18^F-FDG injection. To minimize physiological uptake in muscles and brown fat, the radioisotope is administered in a quiet room with controlled temperature (20–22°C), and beta-blockers (20 mg oral propranolol 1 h before) may be employed in specific situations. In scenarios involving fever of unknown origin or suspected cardiac involvement, a high-fat, carbohydrate-free diet for 48 h, fasting for 12–18 h, or intravenous unfractionated heparin 15 min before ^18^F-FDG injection should be considered (14).

Blood glucose levels below 160 mg/dl before injection are preferable. Although hyperglycemia might not be decisive on the false-negative rate of ^18^F-FDG-PET-CT in the inflammatory context, in contrast to its impact on oncologic indications, a negative correlation exists between glycemia and ^18^F-FDG uptake in blood vessels.

^18^F-FDG-PET-CT acquisition involves low-dose, non-contrast CT for attenuation correction and anatomic reference, performed 90–120 min post-injection (even up to 180 min). Late acquisition is optimal for PET activity detection in GCA, especially in patients already treated with glucocorticoids, as it enhances the vascular wall-to-blood pool activity ratio, improving precision and spatial resolution (15–17).

Depending on local resources and practices, contrast-enhanced CT may be used as modern PET/CT systems allow for CT angiography immediately post-PET acquisition, offering an excellent anatomic assessment in a single modality. This procedure is optional but beneficial for detecting stenosis and characterizing aneurysms; its validity for detecting arterial abnormalities has been proved in one study focused on the evaluation of the superficial temporal artery in GCA (18). Contrast-enhanced CT for attenuation correction can be employed in the venous or equilibrium phase (e.g., delayed acquisition).

Duration of the examination process is 2–3 min per bed, even shorter with digital scanners. A whole-body study covering the vertex to the knees, with the patient in a supine position and arms alongside the body, is recommended. Optionally, the acquisition may be extended to the feet, although the low spatial resolution of ^18^F-FDG-PET-CT for vessels lesser than femoral arteries should be taken into account (19).

The recommended intravenous dose is 2–3 MBq/kg. Corticosteroid treatment may decrease ^18^F-FDG uptake; thus, it is recommended to start treatment after performing ^18^F-FDG-PET-CT, unless ischemic complications are imminent (especially in suspected ocular or temporal arteritis). Performing ^18^F-FDG-PET-CT within 3 days of initiating corticosteroids is an alternative, as sensitivity was proven to be unaffected after administration of a daily dose of methylprednisolone 60 mg (20). As observed by Nielsen et al. (20), after a 10-day course of treatment there is almost a 30–40% reduction in vessel FDG uptake and a 60% decrease in the sensitivity of 18F-FDG-PET-CT for diagnosing LVV. Limited data exist for the 3- to 10-day window, and adherence to the 3-day timeframe is currently recommended (21).

However, a late ^18^F-FDG-PET-CT (beyond the first 10 days of treatment) can often be informative. Narvaez el al. observed that ^18^F-FDG-PET-CT positivity in new-onset GCA patients treated with high-dose oral glucocorticoids was 54.5% in the first two weeks, 38.5% in those treated for 2 to 4 weeks, and 25% in those treated for 4 to 6 weeks. Boluses of intravenous glucocorticoids can distort PET-CT results since the first endovenous bolus of 125 mg (22). Corticosteroids increase hepatic ^18^F-FDG uptake, impacting liver assessment and visual uptake scoring; and may also distort the results of the diagnostic biopsy (23).

There might be a dose-related and duration-related effect of corticosteroid treatment on ^18^F-FDG-PET-CT diagnostic performance. A study comparing different treatment courses found that patients with a positive ^18^F-FDG-PET-CT result for vasculitis were treated with significantly lower doses and lengths of corticosteroid treatment (24).

Long acquisition time, combined with the use of diagnostic scales (see section “2.5 Diagnostic scales”) may decrease the number of false-positive assessments of ^18^F-FDG-PET-CT, also increasing inter and intra-observer agreement.

### 2.3 Diagnostic performance

It is important to emphasize the growing significance of ^18^F-FDG-PET-CT in the diagnosis of LVV.

Previous recommendations (25) discouraged the use of ^18^F-FDG-PET-CT for the assessment of cranial arteries, as evidence regarding the visibility of these vessels with this technique was limited. However, since several studies now support its use for the diagnosis of temporal arteritis, ^18^F-FDG-PET-CT has been included in the new diagnostic criteria for LVV, to the extent that, in many cases, biopsy is no longer necessary (11, 12, 26–28). This modification of diagnostic criteria aims to promote early detection of vasculitis in order to prevent structural damage or long-term sequelae, such as visual loss in GCA or severe focal arterial stenosis in TAK (29).

In their meta-analysis of 400 patients with LVV, Lee et al. (30) observed an overall pooled sensitivity of ^18^F-FDG-PET-CT for diagnosis of 76% and a specificity of 93%. Notably, the sensitivity was higher for GCA compared to TAK, with values of 83% for sensitivity and 90% for specificity (30).

Altered uptake in atherosclerotic blood vessels, particularly in the elderly and at the ilio-femoral arteries, may diminish the specificity of ^18^F-FDG-PET-CT for LVV. While there may be some overlap between LVV and atherosclerosis, distinct patterns of ^18^F-FDG uptake and the presence of calcifications on CT can ease the differential diagnosis: LVV manifests as a linear, diffuse, circumferential uptake, different from the typical mild, patchy uptake pattern of atherosclerosis (14).

Concerns also arise in the diagnosis or assessment of disease activity in LVV patients with arterial grafts. However, it should be noted that ^18^F-FDG uptake restricted to the graft does not imply active vasculitis, but rather indicates a chronic, low-grade, nonspecific reaction to the graft material (11).

### 2.4 Uptake values and distribution

^18^F-FDG-PET-CT imaging reveals vascular uptake in 83% of GCA patients, especially at the subclavian arteries (74%), the aorta (> 50%) and the femoral arteries (37%) (31). A meta-analysis of 6 studies on ^18^F-FDG-PET-CT’s diagnostic utility for GCA found an overall sensitivity of 80% and a specificity of 89%, with an excellent negative predictive value (88%) (32). Some heterogeneity in the evaluation of a positive result was observed depending on the study and the territory examined; in general, a semi-quantitative analysis of ^18^F-FDG uptakes was performed comparing them with those of other anatomical areas; vessel uptake superior to that of liver was considered an efficient marker for vasculitis. Some studies considered positivity for GCA when aortic uptake was greater than that of the liver, or any uptake was detected in the rest of arteries. Other studies used the semi-quantitative score PETVAS, in which a mean value of 6 was found at the time of diagnosis (see section “2.5 Diagnostic scales” for further information about diagnostic scores).

When comparing both GCA phenotypes, LV-GCA patients, compared to those with C-GCA, exhibit a younger age (68 vs. 75 years; *p* = 0.02) and a longer diagnostic delay (12 vs. 4 months; *p* = 0.006). Despite non-statistically significant, they manifest PMR symptoms and lower-extremity involvement more often (33, 34).

Among patients with PMR, those with subclinical GCA exhibit advanced age, prolonged morning stiffness and a higher prevalence of hip pain. They predominantly display a LV-GCA phenotype. However, patients with PMR in the classic GCA group stick to the C-GCA pattern of involvement (35).

The most prevalent ^18^F-FDG-PET-CT imaging pattern observed in PMR patients is a periarticular uptake, notably in the shoulders (80–100%), hips (70–100%), and sternoclavicular joints (43–93%). A recent meta-analysis identified the uptake in the ischial tuberosities as the most sensitive finding for PMR (sensitivity 85.4%; specificity 70.1%), while the uptake in interspinous processes was the most specific (sensitivity 75.4%; specificity 81.4%) (36).

Taking the ischial tuberosities, interspinous bursae, periarticular hips and symphysis pubis enthesis as the characteristic sites for PMR, one study evaluated the characteristic-site standardized uptake values (SUV) index (that is, the mean SUV index of these sites; SUV index being the ratio between lesional maximum SUV [SUVmax] and liver mean SUV) and yielded an area under the ROC curve (AUC) of 0.93, establishing the optimal SUV index threshold at 1.685 for a sensitivity of 84.6% and a specificity of 92.6%. The probability of PMR surpassed 90% when the characteristic-site SUV index exceeded 2.56 (37).

**FIGURE 1:**
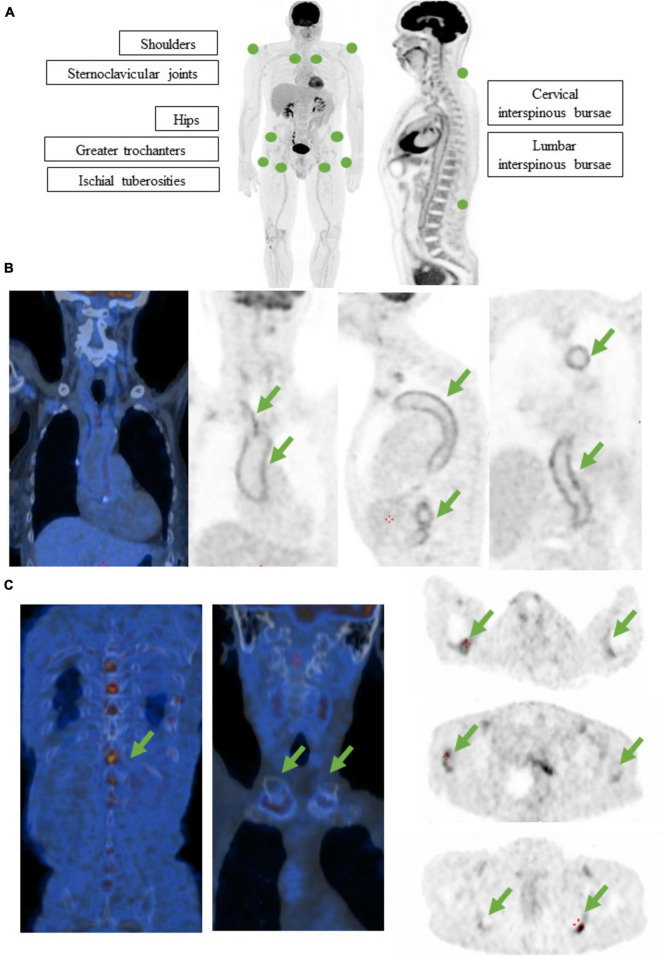
**(A)** Diagram representing the Leuven score for the assessment of PMR probability. A value on a 3-grade visual scale is assigned to each one of the 12 spots depicted, as follows: 0 means no uptake; 1 means less uptake than that of the liver, 2 means equal or more uptake than that of the liver. PMR is considered likely if the total score is equal or above 16 out of 24. **(B)** ^18^F-FDG-PET-CT of an 84-year old female with GCA at diagnosis. Symptoms were asthenia, weight loss, raised inflammatory markers and diffuse bone pain. Notice the intense metabolic activity in aorta and supra-aortic vessels (arrows). Visual score: 3. **(C)** ^18^F-FDG-PET-CT of the same patient. Notice the associated metabolic activity in shoulders, sternoclavicular joints, greater trochanters, ischial tuberosities and lumbar interspinous bursae (arrows). Leuven score: 18. Probable PMR was reported.

Extraarticular uptake is also described in PMR patients as iliopectineal (8–100%), subtrochanteric (71–93%), or ischiogluteal (52–96%) bursitis as well as uptake in the cervical (7–56%) and lumbar (38–87%) spinal processes. Further involvement includes enthesitis and tenosynovitis of the pectineus and long adductor muscles, rectus femoris and biceps femoris, resulting in prepubic, anteroinferior iliac spine, and adjacent ischial tuberosity uptake, respectively (38, 39). Individual uptake assessments lack sufficient diagnostic precision, prompting the development of various scales and algorithms; the Leuven score stands out as the most useful for diagnosis (section “2.5 Diagnostic scales”, Figure 1) (40).

A meta-analysis including TAK patients disclosed a sensitivity of 81% and specificity of 74% for ^18^F-FDG-PET-CT (41, 42). Some features were able to distinguish between GCA and TAK; TAK patients exhibited a higher likelihood of abdominal, carotid and subclavian artery disease, the latter sometimes being focal and restricted to the left subclavian artery (*p* < 0.01). Conversely, GCA patients were more prone to diffuse disease, bilateral axillary/subclavian artery involvement, or minimal disease lacking a discernible pattern (*p* < 0.01). Finally, TAK patients were more likely to have angiographically detectable structural damage, while GCA patients tended to show arterial FDG uptake without associated vascular damage (43).

A comparative overview of LVV and PMR can be found in Table 1.

**TABLE 1:** Overview of large-vessel vasculitis spectrum [refs.: (3, 4, 6, 32, 33, 36, 37, 41, 48)].

	C-GCA	LV-GCA	TAK	PMR
Distribution	Europe	Europe	Asia	Europe
Patients	♀≈ 75 year	♀ > 50 year	♀ < 40 year	♀ > 50 year
Symptoms	• Headache, scalp tenderness, jaw claudication, visual loss. • Fever, anemia, constitutional symptoms	• Fever, anemia, constitutional symptoms • Arm/leg claudication, carotidynia • Vascular bruits, pulse discrepancy	• Fever, anemia, constitutional symptoms • Arm/leg claudication, carotidynia • Vascular bruits, pulse discrepancy	• Shoulder and pelvic girdle pain. • Morning stiffness • Arthritis, bursitis, tenosynovitis • Constitutional symptoms
Structures involved	• Aorta and major branches (aneurysms) • Subclavian arteries • Temporal, ocular arteries	• Aorta and major branches (aneurysms) • Femoral arteries	• Aorta and major branches (stenosis) • Renal, mesenteric, carotid, left subclavian arteries • Coronary arteries	Periarticular involvement of: • Shoulders and hips • Sternoclavicular joints • Ischial tuberosities • Interspinous cervical- lumbar bursae • Symphysis pubis • Anterior inferior iliac spines
Overlapping	• PMR (53%) • LV-GCA (30%)	• PMR (35%)		• GCA (10–40%). Suspected if refractory-atypical PMR. • Subclinical GCA (> 25%), more often LV-GCA.
Preferred imaging diagnosis (EULAR)	1. Doppler-US (temporal and axillary arteries) 2. PET-CT or MRI	1. PET-CT 2. MRI or CT	1. MRI 2. PET-CT	Clinical diagnosis, optional US evaluation of shoulder/hip
^18^F-FDG-PET-CT diagnostic performance	Initial diagnosis: • Sensitivity 80% • Specificity 89% Treatment response: • Sensitivity 78% • Specificity 71%	Initial diagnosis: • Sensitivity 80% • Specificity 89% Treatment response: • Sensitivity 78% • Specificity 71%	Sensitivity 81% Specificity 74%	• Most sensitive: Ischial tuberosities 85% • Most specific: Interspinous processes 81% • Leuven Score ≥ 16: Sensitivity 91% Specificity 98%

C-GCA, cranial giant cell arteritis; EULAR, European Alliance of Associations for Rheumatology; LV-GCA, large-vessel giant cell arteritis; LVV, large-vessel vasculitis; PMR, polymyalgia rheumatica.

### 2.5 Diagnostic scales

Various interpretation criteria for ^18^F-FDG-PET-CT have been proposed. Existing evidence suggests that semi-quantitative parameters may not be superior to a visual grading scale in the routine clinical diagnosis of LVV (44).

A standardized 4-point visual grading scale, based on the comparison between arterial and liver uptake, is recommended as follows: grade 0 for no uptake, grade 1 for lower arterial than liver uptake, grade 2 for similar arterial and liver uptake, and grade 3 for higher arterial than liver uptake. Grade 3 is considered positive for LVV, while grade 2 indicates possible LVV (14, 45). The cranial arteries are evaluated with a 3-point visual grading scale based on the comparison between the arterial uptake and that of the surrounding tissue: grade 0 indicates arterial uptake not above that of the surrounding tissue, grade 1 indicates arterial uptake just above that of the surrounding tissue, and grade 2 indicates arterial uptake significantly above that of the surrounding tissue (14, 46). In cases of active liver disease when hepatic uptake is increased, the uptake of the arterial vessels is compared with that of the vena cava to avoid comparison mistakes.

Additionally, a quantitative composite score, known as the PET vascular activity score or total vascular score (PETVAS or TVAS) is based on a visual grading scale of 7 to 15 arterial segments. This score offers an overall assessment of disease burden with proven robustness and minimal interobserver variability. The PETVAS score may be preferred for evaluating treatment response. In one study, a ROC curve analysis showed that a PETVAS ≥ 10 yielded 60.8% sensitivity and 80.6% specificity to distinguish clinically active from inactive LVV, with an AUC of 0.73 (47).

Regarding PMR, the Leuven Score, developed by Henckaerts et al. in a prospective study, is a semiquantitative evaluation of 12 anatomical landmarks (shoulders, sternoclavicular joints, hips, greater trochanters, ischial tuberosities and cervical and lumbar interspinous bursae). Each one is assigned a value of 0 to 2 depending on the uptake intensity. It has demonstrated optimal sensitivity (91.4%) and specificity (97.6%) at a cut-off point of 16 for clinical diagnosis of PMR (Figure 1). A concise Leuven/Groningen Score, focused on the evaluation of 7 anatomical sites, might perform equally well, although further validation is required (39, 48, 49).

A summary of the most relevant diagnostic scales can be found in Table 2.

**TABLE 2:** Summary of the most relevant diagnostic scales for LVV and PMR [refs.: (39, 45, 47–49)].

	Meller score	Leuven score	Leuven-Groningen score	PETVAS or TVAS	SUVmax aorta	SUVmax most active cranial artery	SUVmax aorta to liver ratio*
Disease	LVV	PMR	PMR	LVV	LVV	C-GCA	LVV
Type	Visual	Visual	Visual	Visual	Semi-quantitative	Semi-quantitative	Semi-quantitative
Preferred application	Diagnosis and activity	Diagnosis and activity	Diagnosis and activity	Activity and Treatment monitoring	Diagnosis and activity; Patients under GCs	Diagnosis and activity	Diagnosis and activity
Cut-off value	2–3	16	7–8	10	3.12	5	1.03
Diagnostic performance	Grade 2: Sensitivity 100% Specificity 51% Grade 3: Sensitivity 83% Specificity 91%	Sensitivity 91% Specificity 98%	Value 7: Sensitivity 97% Specificity 93% Value 8: Sensitivity 93% Specificity 95%	Sensitivity 61% Specificity 81%	Sensitivity 83% Specificity 73%	Sensitivity 79% Specificity 92%	Sensitivity 72% Specificity 92%
Pros	Easy to apply. Great diagnostic values in grade 3	Standardization of PMR findings	Easier than Leuven Score	Objective and reproducible. Overall assessment of LVV	Objective and reproducible	Objective and reproducible	Objective and reproducible
Cons	Subjective	Time-consuming	Needs further validation	Time-consuming	Absolute, non-relative values	Absolute, non-relative values	Time-consuming. Added value to Meller score is doubtful

*Other semi-quantitative, target-to-background ratio (TBR) approaches are vascular/liver ratio, vascular/lung ratio, vascular/blood pool ratio and arterial/venous ratio. C-GCA, cranial giant cell arteritis; GC, glucocorticoid; LVV, large-vessel vasculitis; PETVAS, positron-emission-tomography vascular assessment score; PMR, polymyalgia rheumatica; SUV, standardized uptake value; TVAS, total vascular assessment score.

### 2.6 Prognostic value

In patients with LVV, assessing both the intensity and extent of vascular FDG uptake at diagnosis can predict their clinical outcome (50). Prior research has hinted at the correlation between aortic ^18^F-FDG uptake at the time of diagnosis and an elevated long-term risk of aortic aneurysm development (51). Other studies have identified an association between FDG uptake at the thoracic aorta and late thoracic aorta volume (*p* = 0.039); and between a positive ^18^F-FDG-PET-CT scan and an increased likelihood of aortic complications (*p* = 0.004) over a 5-year timeframe (52). Specific guidelines on aortic sequelae monitoring in LVV are required.

Future investigations are imperative to explore the utility as a prognostic indicator for PMR.

### 2.7 Assessment of treatment response

The value of imaging techniques for disease monitoring is becoming increasingly important, moreover when several of the recent treatments for LVV directly influence acute phase reactants, rendering them unreliable for the assessment of disease activity (53). ^18^F-FDG-PET-CT shows promising results in evaluating treatment response in GCA and PMR, through assessment of metabolic activity and vascular structural changes (54). Some studies also suggest that late-acquisition PET-CT may be useful in detecting activity, even in completely treated patients (15–17).

Despite ^18^F-FDG-PET-CT is not being routinely recommended for treatment monitoring in GCA (55), most studies demonstrate a decline in both the extent and intensity of ^18^F-FDG uptake during treatment. A meta-analysis has shown that ^18^F-FDG-PET-CT provides a moderate sensitivity of 78% and a specificity of 71% in discerning active from quiescent LV-GCA during treatment (56). The impact of treatment on arterial wall uptake is not exclusive to glucocorticoid therapy; analogous reductions have been observed in GCA patients treated with methotrexate and anti–interleukin (IL)-6 therapy such as tocilizumab and sarilumab (57, 58).

The prevailing consensus states that a reduction in uptake intensity exceeding 20% and/or a decline in the extent of FDG uptake can be considered indicative of a therapeutic response (59). Nonetheless, the use of ^18^F-FDG-PET-CT is controversial as residual activity is often observed despite complete clinical and biological response; although high-dose glucocorticoid treatment exerts substantial effects on ^18^F-FDG uptake after 10 days, persistent arterial wall uptake may last throughout treatment-induced remission, extending up to 6 months post-initiation (14, 56, 60). Multiple potential explanations have been proposed for this phenomenon, such as low inflammatory vascular remodeling, the chronic vasculitis phase, angiogenesis, chronic hyperglycemia and atherosclerosis plaques.

As a result, there is currently no consensus on the optimal timing for performing post-treatment ^18^F-FDG-PET-CT. Blockmans et al. (61) conducted baseline ^18^F-FDG-PET-CT imaging at 3 and 6 months following corticosteroid treatment: the total vascular score decreased from 7.9 ± 5.5 at baseline to 2.4 ± 3.5 at 3 months (*p* < 0.0005), with no further reduction at 6 months.

A recent meta-analysis of cross-sectional studies suggested that ^18^F-FDG-PET-CT could detect relapsing/refractory disease with a sensitivity of 77% and a specificity of 71% (62).

Experience regarding the role of ^18^F-FDG-PET-CT in monitoring PMR treatment is limited, as clinical evaluation typically guides treatment response assessment. Analogous to arterial wall ^18^F-FDG uptake in LVV, studies in PMR patients demonstrate a reduction, though not necessarily normalization, of ^18^F-FDG uptake at the shoulder, pelvic girdle, and interspinous bursae during treatment-induced remission (63). No study has yet investigated whether disease activity can be monitored with ^18^F-FDG-PET-CT in PMR patients treated with glucocorticoid-sparing agents.

### 2.8 Other imaging techniques and future directions

Prior research has revealed comparable effectiveness of ^18^F-FDG-PET-CT and extended vascular ultrasound for GCA diagnosis; the former excels in detecting aortic or vertebral vasculitis while ruling out alternative diagnoses, whereas the latter, more widely accessible, adds value to the identification of temporal and popliteal vasculitis, and to the measurement of the severity of stenosis and flow direction (64, 65). Likewise, similar findings have been reported regarding the diagnostic accuracy of CT angiography (CTA) compared to ^18^F-FDG-PET-CT, even when slice thickness tends to be greater in ^18^F-FDG-PET-CT scans.

^18^F-fluorodeoxyglucose positron emission tomography-magnetic resonance imaging (^18^F-FDG-PET-MRI) is a good candidate for the evaluation of LVV and PMR. Its outstanding contrast resolution allows precise anatomical localization of PET tracer uptake while avoiding radiation exposure (this is especially applicable to younger TAK patients); possibly improving evaluation of narrow cranial arteries, characterization of vessel wall inflammation and organ assessment, including cerebral parenchyma and bone marrow. One study evaluating target-to-background ratios (TBRs), maximum standardized uptake values (SUVmax), and visual scores found robust correlations between ^18^F-FDG-PET-MRI and ^18^F-FDG-PET-CT (*r* = 0.92, *r* = 0.91, *r* = 0.84; *p* < 0.05) (66). However, further studies are imperative to assess the utility of ^18^F-FDG-PET-MRI in the evaluation of LV and PMR.

Another interesting topic is the utility of delayed-acquisition ^18^F-FDG-PET-CT (at 150–180 min post-injection) in patients with LVV under treatment with corticosteroids, and even more so with biologic agents. Late acquisition could be useful to identify false negative cases, thus potentially rescuing patients who might otherwise be overlooked (17, 67).

Finally, as controversy exists regarding persistent ^18^F-FDG uptake after treatment, the use of novel targeted PET-CT tracers could serve as an alternative for doubtful cases; further studies are needed to assess value of T-cell, macrophage or fibroblast specific radiotracers, such as the fibroblast activation protein inhibitor (FAPI) (68).

## Author contributions

JC-C: Writing – original draft. NG-L: Writing – original draft. VC-M: Writing – review & editing. BL-F: Writing – review & editing. SC: Writing – review & editing. VR-L: Writing – review & editing.
